# Peer-to-peer sharing in public health interventions: strategies when people share health-related personal information on social media

**DOI:** 10.1080/17482631.2024.2367841

**Published:** 2024-06-26

**Authors:** Jens Lindberg, Anna Sofia Lundgren

**Affiliations:** a Department of Social Work, Umeå University, Umeå, Sweden; b Department of Culture and Media Studies, Umeå University, Umeå, Sweden; c Centre for Demographic and Ageing Research, Umeå University, Umeå, Sweden

**Keywords:** Social media, social media sharing, social media dilemmas, health information, personal health data, public health, health interventions, older people

## Abstract

**Purpose:**

As sharing on social media has become an integrated part of everyday life, health and public health actors have started to show interest in the potential of people’s peer-to-peer sharing of health-related personal information (HRI) for health interventions. In this article we focus on how people make sense of sharing HRI on social media.

**Methods:**

Twenty-two people between the ages 40 and 60 who had taken part in a regional health intervention were interviewed. Using theories about social media sharing, we explore their understandings and negotiations about whether, how much, and how to share HRI and discuss the results in relation to peer-to-peer sharing as a strategy in interventions.

**Results:**

We identified three aspects that were perceived as particularly risky: loss of control, effects on identity, and affecting others negatively, along with strategies that were used to manage risks in practice: avoiding sharing, allocating, and embedding HRI.

**Conclusions:**

By allocating and embedding HRI, people can unlock motivating affordances for health work. However, strategies to manage risks can also be counterproductive. For actors to provide equality in health promotion, initiatives that include social media sharing need to be mindful of the sometimes counterproductive effects this may have on people’s engagement.

## Introduction

Over the course of the last two decades, social media sharing has increasingly become what has been described as an integrated part of people’s everyday lives, with people sharing content from both their professional and private lives. Based on studies suggesting that the supportive functions of online social networks can benefit health- and lifestyle-related activities at the individual, community, and population levels (Chen et al., 2019; Le et al., 2022; Oeldorf-Hirsch et al., 2019; Zhang et al., 2020), the fields of health and public health have been quick to recognize peer-to-peer sharing on social media as a possibility (Abroms, 2019). Following such developments, interest in what people do with their health-related information (HRI) has grown significantly (Karampela et al., 2018) and peer-to-peer sharing of HRI is increasingly being suggested as a method to be used as part of health and public health interventions (Chen & Wang, 2021). Hence, people are becoming encouraged to engage not only in online health information-*seeking* activities, but also in online health information-*sharing* activities (Li et al., 2018).

Relating to this, research has identified various arguments for sharing HRI on social media. It has been claimed that people are open to sharing HRI in online environments that are perceived to be safe (Kim & Choi, 2019) and that posting (and consuming) HRI helps to produce feelings of motivation in people’s health work (Lin et al., 2018). Research has also identified arguments against sharing HRI on social media, such as fear of negative consequences and experiences from exposing oneself publicly on social media (Ahmed et al., 2019; Le et al., 2022; Pagoto et al., 2019). There are, however, suggestions that there might be differences among populations in terms of how the sharing of HRI is made sense of and practised, and that the role attributed to social media may be based on studies of younger people. In the area of digital care, internalized social and cultural notions about privacy and sharing seem to vary among different groups of people (Schairer et al., 2019). For example, younger people appear more open than older people to disclosing personal information online (Newman et al., 2021). In empirical studies targeting groups aged 55 and older, it has been stated that many older people do not see the point of engaging with social media (Jung et al., 2017)—interactions on social media are seen as trivial and self-centred (Lüders & Brandtzæg, 2017). Older people also tend to be more concerned about online privacy, use only limited functions within social media systems, or fail to use social media at all due to a perceived lack of skill (Wilson et al., 2023). As concluded by Newman et al. (2021), it appears that older people tend to use social media to maintain existing social networks rather than interacting with different audiences in a variety of ways. In research about the sharing of HRI on social media, these social, cultural, and generational aspects of social media sharing have not been sufficiently explored. So far, empirical studies have almost exclusively focused on the supposed *benefits* of people’s sharing practices by, for example, exploring causal relations between social media sharing and positive health outcomes (Chen & Wang, 2021) and finding ways to strengthen the overall efficiency of health-related interventions by using people’s social media sharing (Giustini et al., 2018; Le et al., 2022; Zhang et al., 2020). Many studies thus employ de-contextualized approaches (Rui, 2023) and rely on behavioural and cognitive theories that focus on people’s internal and external motivations (Le et al., 2022).

Given the increased interest in using the peer-to-peer sharing of HRI as a tool in interventions (e.g., Fung et al., 2020; Keller et al., 2014), it is vital that its workings and consequences are discussed more thoroughly (Smailhodzic et al., 2021). To fully grasp the significance of using people’s sharing practices as part of health and public health initiatives, Le et al. (2022) emphasize the need for empirical explorations of the social and emotional aspects that are evoked when people engage in online sharing. This could include, for example, studies of people’s day-to-day *uses* of social media (Zhang et al., 2020), their *sensemaking* around collecting and sharing personal data digitally (Lupton, 2021a), and potential *differences* between groups of people in terms of approaches to sharing HRI. Such aspects are indeed key to furthering our understanding of the broader processes in which social and technological imperatives to share HRI are included in health and public health initiatives, and which may certainly be understood and managed differently by different people.

In this article, we focus on people’s sensemaking about peer-to-peer sharing of HRI on social media. Inspired by Cino and Formenti’s (2021) approach to social media sharing, the important research questions were: What dilemmas did study participants articulate when reflecting about sharing HRI on social media? What ways of resolving those dilemmas were described? What were the social and technological underpinnings of the dilemmas and negotiated solutions? We argue that there is much to learn from the processes involved as people choose if, and if so how, to share HRI online. Still, our intent is neither to help facilitate nor to dismiss social media sharing as a tool for health and public health actors. By exploring people’s situated negotiations, it is possible to critically discuss the sharing of HRI in relation to social media sharing as an emerging strategy in public interventions.

This study was conducted as part of a collaborative research programme (Ng et al., 2021) focusing on online aspects of the Västerbotten Intervention Programme, a public health initiative in the region of Västerbotten, Sweden (see Norberg et al., 2010). Given the increasing interest in social media sharing during health interventions, we believe that our results and discussions are relevant to actors looking into peer-to-peer sharing of HRI as a strategy in health and public health work.

## Theoretical framework

Studies of peer-to-peer sharing on social media unequivocally show that it is closely connected to considerations related to identity. In this age of social media, Lupton (2021b) argues, HRI is an essential part of people’s digital identity constructions. This forces them to make strategic choices about what and how to share—even though many have only very limited knowledge about how to ensure online privacy (Kwok Choon, 2018). Such choices are not without difficulties. Kwok Choon (2018) stresses that values often conflict in social media sharing, and Liao (2019) has noted that the sharing of HRI seems particularly vulnerable to tensions between conflicting motives, such as achieving desired self-presentations and maintaining privacy. With this in mind, Cino and Formenti (2021, p. 492) have conceptualized the choices that people are confronted with as “social media dilemmas”—the simultaneously mundane and perplexing situations that people repeatedly find themselves in when they “go about balancing the pros and cons of whether and how to share” personal information about health and lifestyle on social media. How social media dilemmas are identified and understood is influenced by the social, cultural, and technological contexts in which people are situated (Costa, 2018; Kennedy, 2016) and which enable but also confine people’s approaches to those dilemmas (Davis & Jurgenson, 2014; McVeigh-Schultz & Baym, 2015). What constitutes a dilemma is an empirical question—dilemmas must be identified in relation to people’s situated experiences.

Despite the sometimes difficult considerations involved, there are emerging social and cultural discourses that encourage, even demand, the sharing of quite personal information online (John, 2017). Fulton and Kibby (2017) have discussed the contemporary encouragement of online sharing in terms of a normalized surveillance, where people increasingly accept having friends, colleagues, and family with whom they have differently close relationships having access to relatively personal information. Such encouragements to share also follow from what have been coined the “affordances” of social media technologies (Hutchby, 2001; Ronzhyn et al., 2023): “the mutuality of actor intentions and technology capabilities that provide the potential for a particular action” in people’s use of social media (Majchrzak et al., 2013, p. 39). Overall, the widespread “public-by-default” setting of contemporary Internet culture and social media technology makes sharing not primarily an individual choice, but a socio-technological imperative (Lupton, 2016; Van Dijck, 2013). Not least, such encouragement is undertaken by the social media companies, which have a vested interest in people sharing personal information. For example, Friesen (2017) describes how Facebook responded to the decreasing numbers of user-authored posts by adjustments to increase the visibility of that type of post. But sharing is also promoted by public actors. As noted by Li et al. (2018), people are increasingly being urged to share their HRI for the cause of individual and public health.

Empirical studies have effectively shown that the ongoing entanglements between social and technological conditions are key to understanding the production of feelings such as motivation for lifestyle change (e.g., Berg, 2017; Lindberg & Lundgren, 2022). However, if social media sharing is comprehended as inherently problematic, there is reason to believe that such entanglements also produce feelings that might reduce motivation. We therefore argue that there is a need to further explore the perceived dilemmas involved in the practice of sharing HRI online, to gain a better understanding of the situated comprehensions and negotiations involved in such sharing, and to explore what this might mean for health and public health actors, looking at peer-to-peer sharing on social media as a tool for future interventions.

## Methods

Below, we begin by describing the process of selection for the study, including general information about the study participants and important research ethical considerations. We then present the procedures employed to gather the research material, and the approaches used to analyse it.

### Selection

Twenty-two people were included in the study. They had all taken part in the Västerbotten Intervention Programme (VIP) (Norberg et al., 2010), which means that they had recently undergone a health examination that included nine measures: physical activity and sedentary behaviour, food habits, tobacco use, alcohol consumption, BMI and waist circumference, blood pressure, blood glucose, cholesterol level, and perceived health. They had also had their test results communicated to them during a “health dialogue” with a nurse in which they had discussed the results and any possible changes in lifestyle that were deemed necessary.

The selection of participants was informed by a desire to include both women and men, living in both rural and urban parts of the region Västerbotten in the north of Sweden. Altogether 13 women and nine men were interviewed. Around half of the participants were living in a city or in close proximity to a city. The other half were living in small towns or communities further away from a city. The medical encounter included in the VIP intervention is offered to the population of Västerbotten every ten years—at ages 40, 50, and 60. The selection included participants mainly distributed around the ages of 40 and 60. Out of 22 study participants, ten were around 40 years of age, ten were around 60 years of age, and two were around 50 years of age.

Participants who accepted the invitation to be interviewed were included regardless of their experience of using social media. However, all study participants turned out to have some experience of using social media systems, but to very different extents. Most of the participants used Facebook regularly. Many also used Instagram, sometimes in combination with Facebook. Supplementing Facebook and Instagram, several participants used messaging applications like Snapchat, WhatsApp, and Messenger, but often only in relation to family and friends. The younger participants were generally more avid users of social media in the sense that they used social media systems more frequently, and also a greater variety of systems in combination.

The study was approved by the Swedish Ethical Review Authority (Dnr: 2019–02924; 2020–02985) and guided by research ethical principles as defined by The Swedish Research Council (2017). Our research ethical stance was also influenced by particular contextual conditions. Many of the study participants live in small towns and communities in the region of Västerbotten, and the study touches upon sensitive topics such as health, lifestyle, and online privacy. To minimize the risk of identification, we provide only general information about study participants in this article.

### Research material

The gathering of research material for the study was conducted during 2020–2021. Fieldwork was originally designed for semi-structured, face-to-face interviews. However, due to restrictions following the COVID-19 pandemic, these methods had to be altered to use more remote approaches as we were not able to meet physically with the participants. The first five interviews were conducted face to face; then, after restrictions set in, interviews were conducted via telephone and Zoom. When using Zoom, interviewees decided whether they wanted to have cameras on or not. However, only sound was recorded. The format of the interviews was guided by insights from scholars conducting qualitative inquiries during the COVID-19 pandemic (e.g., Howlett, 2022; Watson & Lupton, 2022). This included, for example, keeping interviews shorter and more focused than would probably have been the case if they had been conducted during physical encounters (Janghorban et al., 2014). The face-to-face interviews were generally longer, lasting up to two hours. The longest telephone/Zoom interview lasted for one hour and 40 minutes, but the majority were just over an hour long. Both face-to-face and telephone/Zoom interviews were transcribed verbatim. Informed consent was obtained in writing for face-to-face interviews and verbally for telephone/Zoom interviews.

The interview guide for the study revolved around five general themes: everyday use of social media and different social media systems; perceptions of sharing and consuming health-related information on (and off) social media; the role of social media in participants’ health work; visualization and datafication of health-related personal information; and matters of privacy in the context of social media. During the semi-structured interviews, the study participants were also asked questions about their VIP experiences in general. This included questions about the health examination (when samples and measures were taken, and a questionnaire was filled out by the participants) and the following health dialogue (when participants met with a VIP nurse who used a star-shaped visualization technique to communicate the results of the examination). Their recent participation in the VIP intervention seemed to make it easy for participants to recall the medical encounters and how they had experienced having their health data explained to them.

Combined with the semi-structured character of the interviews, the general nature of the themes enabled participants to reflect freely and to immerse themselves in aspects that were not part of explicit questions (Gubrium & Holstein, 2003). The semi-structured approach to interviewing did, however, uncover limitations related to the strategic decisions imposed by the COVID-19 pandemic. Shorter telephone and Zoom interviews made it difficult for some participants to elaborate upon abstract and sensitive matters (cf., Hall et al., 2021). To compensate for this, the interviewers made special efforts to follow up on questions that seemed abstract and sensitive, and difficult for participants to answer.

Nevertheless, during the interviews, most participants talked a great deal about their HRI, ranging from detailed medical information (e.g., blood pressure, cholesterol level, and body-mass index (BMI)) and lifestyle-oriented behaviours (e.g., diets, physical activity, and tobacco and alcohol consumption) to more abstract aspects, such as their relation to their own HRI, their perceived motivation to enact lifestyle changes, and their thoughts on sharing different kinds of health-related personal information on social media. It was clear that people related differently to the various aspects, and what one person considered to be private or sensitive information was not so for another. The perceived dilemmas were not only related to the health information as such, but also to the anticipated effects of sharing them on social media. Generally, it was when discussing what they considered private and sensitive that participants expressed hesitation regarding social media sharing. But hesitation also included participants’ experiences of, and approaches to, social media, and their personal feelings towards the socio-technological imperative of social media sharing at a general level (Lupton, 2016; Van Dijck, 2013).

### Analysis

Analyses of the research material were structured with inspiration from Cino and Formenti’s (2021) dilemmic approach to social media sharing and Braun and Clarke’s (2019) approach to thematic analysis. This combination resulted in an abductive analytical process in which we read through the transcribed interviews several times to identify aspects that appeared to be particularly meaningful to study participants in the context of sharing HRI. In order to explore dilemmas, we were also attentive to conflicting, even contradictory, approaches to sharing.

In this ongoing process of identification and interpretation, the first step was to *identify* and describe the perceived dilemmas that the study participants articulated. This meant that we focused on instances when participants began to hesitate or negotiate different sharing approaches by discussing the perceived risks involved in the sharing of HRI.

In a second step, we identified and described the ways in which the participants *resolved or suggested solutions to* the dilemmas—including different strategies for how to share or not to share. This also included the arguments involved when participants reflected upon their own sharing practices.

Finally, in the discussion section, we theorize and discuss the social, cultural, and technological underpinnings of what we suggest could be important dilemmas and solutions in people’s sharing of HRI. This also entails reflecting upon the results and their potential consequences for an increasing number of interventions that might use peer-to-peer sharing of HRI as a tool in health and public health work.

## Results

It was clear that many of the participants in this study approached social media as an inherent part of their everyday social life (Fotopoulou, 2018) and that sharing personal information was a pivotal part of their social media engagement (Ham et al., 2019). All the participants had experience of using social media platforms to share textual and visual content. Some talked at length about their experiences of sharing HRI to do with physical exercise, and then highlighted that this sharing worked to gain motivation for health work. In such stories, social media sharing seemed to work almost as a public promise that would tie them to their health-related practices in a positive sense, and could result in important positive feedback in the form of likes and encouraging comments. However, despite surfacing in the interviews as a potential perk of social media HRI sharing, most of the participants would still prefer *not* to share their HRI on social media due to the risks that they associated with the practice.

Below, we describe the issues that emerged as dilemmas in the participants’ accounts. We then go on to describe their suggested solutions to these dilemmas. Finally, we discuss the social and technological underpinnings of the suggested dilemmas and solutions, and the potential consequences of the results for emerging initiatives and interventions.

### Dilemmas

Despite living in a culture where online sharing has been described as a socio-technological imperative (Lupton, 2016; Van Dijck, 2013), and agreeing to a certain extent with the mundaneness of sharing practices, the participants’ fear of “oversharing” (Kennedy, 2018) was an overwhelmingly dominant theme in the interviews. This theme was held together by the view that their social media practices would instantly reflect upon themselves as individuals, and by the notion that the sharing of HRI was negative, both for individuals and for society in general (Buehler, 2017). The fear of oversharing consisted of two interconnected dilemmas that stood out as frequently recurring: loss of control over intimate data and effects on identity. To these, a third dilemma could be added: the risk of affecting others negatively. All three were interlaced with morally charged notions related to the transgression of the boundary between private and public, and of the idea that what was shared had real consequences.

#### Loss of control

When reflecting upon the ability to share HRI on social media, participants consistently related to social media as spaces where everything is shared and public (Jensen, 2007). The risks associated with the sharing of HRI were often articulated in terms of privacy and connected to information that was termed “private” and “sensitive”. There was a lot of variation in interviews about what was described as private and sensitive information, but participants generally tended to describe it in terms of detailed medical data, such as blood-pressure, body mass index, cholesterol, or blood fat. Such health data were all included in the VIP intervention in which they had taken part. But information deemed private and sensitive would also include more lifestyle-oriented matters, such as information about dietary habits, physical activity, tobacco use, and alcohol consumption, or questions about mental health.

While participants generally approached social media sharing as an inherent and even joyful part of everyday social life (Fotopoulou, 2018; Ham et al., 2019), attitudes to sharing HRI were strongly influenced by the perceived delicacy of information related to personal health and lifestyle. Compared to other types of personal information that were shared, information connected to personal health was seen as distinctly more sensitive in nature (Frost et al., 2014; Karampela et al., 2018; Schomakers et al., 2019; Zhang et al., 2018). This sensitivity was in turn closely connected to notions of privacy, which were also expressed as a major reason for not wanting to share HRI.

Many participants had experiences of sharing HRI “analogically” with family, friends, and even colleagues, but did not want to share it on social media, either because they felt uncertain about who would be able to see it or due to a general feeling of losing control over the information. One participant in her 60s, who was otherwise very active on social media, would happily post “everyday nonsense” but never detailed information related to personal health: “I don’t want someone unknown [to see my HRI], I don’t post anything that I think is private that I don’t want anyone else to see”. Others claimed that it was because one cannot control the context on social media or what will become of the information:

Because there [on social media] I think you’re so vulnerable. I mean, you feel (…) uncomfortable. (…) When you disclose something, first of all, you never know, well, what will become of it [on social media]. (Woman in her 60s)

It seemed as though the conception that social media content was widely and openly distributed, combined with the perceived sensitivity of HRI, created uncertainties about where, and with whom, HRI would end up if posted on social media.

#### Effects on identity

Closely connected to the feeling of losing control over information were the risks associated with *identity*. The worries about privacy and exposure were mostly tangled up with participants’ fears of being judged. Such fears were sometimes related to the notion of social media sharing as a form of public promise and were humorously described as a fear of having one’s possible failure displayed publicly on social media. Fears were also related to what participants would *become* if their social media profile and, consequently, other users’ social media feeds, were to be filled with their personal HRI. It was clear that social media profiles were seen as an important forum for self-presentation, and participants asked themselves whether presenting themselves via HRI sharing was really compatible with how they wanted to be perceived. However, and more commonly, the fears were related to either the unspecified risks of outing health-related problems that one’s social media followers and friends were not aware of, or the risks of becoming associated with *boasting* or even *bragging*. “It feels like I’m boasting!” was a recurring statement used to explain why participants did not want to share their HRI online, regardless of whether it concerned data such as cholesterol levels or just information about exercise or a new dietary goal.

The fear of boasting or bragging was informed by a general view of social media sharing that connected it with self-aggrandizing endeavours as well as with a lack of boundaries—the inability to draw socially important lines between private and public. Such an inability was in turn related to attention-seeking and unambiguously charged with negativity. The risks of being read as boastful, or indeed as lacking boundaries, increased with the perceived uncertainties about who would be able to see one’s posts and how they would be contextualized. A participant in her 40s reflected on the risks that came with social media followers having different points of view: “Maybe those who take care of themselves a little less (…) perceive it as ‘Now she’s going to post how perfect she is’”. So, while participants often felt confident that most of the friends and followers in their digital networks would react positively, many dwelled on the risks that someone might react negatively.

#### Affecting others negatively

Some participants were also hesitant about sharing specific information, such as information about body weight and dietary habits, due to how they thought it would affect others. This hesitancy built on a belief that certain kinds of HRI could affect the ways in which others felt about their own bodies and lifestyles, potentially sparking problematic feelings and behaviours. One participant described first-hand experience of this, because a person close to her had suffered from anorexia, and the participant knew that information about diets or weight could easily trigger the illness. For this participant, personal desires to post and receive responses about physical activities were complicated by a fear of sharing content that would have a negative effect on friends or others. Another participant, who actively restricted his social media presence, discussed similar matters. He described his belief that HRI that was posted could affect different audiences on social media:

They might feel bad about [my HRI]. (…) They work really hard, but nothing happens and then suddenly I come along and show that, “Well, I’m perfect and I don’t have to do much”. That could go wrong. (Man in his 40s)

This dilemma of having an effect on others builds on the same premise as the arguments in favour of HRI sharing as part of health and public health interventions. They both build on a view of social media as reaching beyond its digital confines and having the potential to *do* things—to make people act and feel things.

The three interconnected dilemmas described by participants—losing control over intimate data, having one’s identity affected, and having an effect on others—all emanated from a view of social media as setting in motion a series of social relations and processes over which the participants had neither knowledge nor control (Vitak, 2012), and which spanned both online and offline worlds. The latter in particular seemed to influence the participants’ choices *not* to share (some aspects of) HRI on social media. All aspects were entangled in morally charged notions of the relationship between private and public, and the dangers that would potentially emanate from transgressing the boundary between them. Interestingly, and similarly to Van Dijck’s (2013) findings, participants recognized the imperative to transgress boundaries between private and public as a function that is inherent in social media technologies. Nevertheless, they primarily discussed the requirement not to overshare as an individual responsibility and oversharing as an expression of (unwanted) individual characteristics (Kent, 2018).

While the dilemmas described above led participants to hesitate about sharing HRI, decisions about sharing or non-sharing were seldom binary. As noted by Salte (2023), people’s responses to the sharing of personal information on social media should not be understood as *either* accepting *or* resisting, and the participants engaged in different types of solutions to the perceived dilemmas.

### Resolving dilemmas

The dilemmas identified above were largely dealt with through participants’ concrete decisions about what to share and why, as well as practical strategies for managing the perceived risks. According to Cino and Formenti (2021), such solutions, and the legitimizing arguments that accompany them, can offer further perspectives on the dilemmas.

#### Avoiding sharing HRI

Many participants were reluctant to share HRI, and some chose not to do so at all. This was a strategy that led to participants losing out on the perks, in terms of encouragement, that sharing can provide. It supported a critical stance towards social media culture, where online identities constitute the foundations for living.

Many nonetheless described situations in which they might consider sharing such information anyway. They seemed to have relatively clear ideas about how to accomplish such sharing. Two strategies that seemed to resolve the perceived dilemmas were particularly common: to allocate health-related information, and to use strategies that increased their control over the ways in which their HRI content would be read by others. In everyday sharing, these solutions were often intertwined in order to strategically restrict or control information. Below, they are described separately for clarity.

#### Allocating HRI

Instead of dismissing the sharing of HRI altogether, a common strategy for controlling information that was perceived to be sensitive and private was to allocate such information to social media spaces where not all friends/followers were allowed. This could often imply having multiple accounts or creating lists within the platforms to keep audiences apart:

I have like two accounts [on Instagram], one that’s more open to everyone, where you can post a nature picture when there’s something beautiful or something like that, and one that’s really just my closest circle. (Woman in her 40s)

Other participants used settings to exclude followers from seeing certain content. Another woman in her 40s explained how different “groups” could be created and how, within those groups, different content could be shared. One group that this participant had created included only family and close friends and, in that group, this participant would regularly share information about health and lifestyle to elicit motivation for health-related work.

The reason given for allocating HRI information in this way was primarily a fear of exposing sensitive information to the “wrong” audience—hence addressing the dilemmas of control over HRI and its effects on identity. What constituted the wrong audience included both concrete followers and friends with whom participants did not have close relationships, and also a vague idea that someone unknown to them could access their information. In a sense, it was a question of risky “context collapse” (Marwick & boyd, 2011)—something that is crucial for LGBTQI+ people and others who may risk sanctions should personal information fall into the wrong hands (Duguay, 2016)—and the strategy was directed towards controlling who could see what personal information. The older participants in particular, although this concern was not confined to them, expressed hesitancy about who could access information that was shared on social media sites. Their strategies made it possible to keep different social domains apart in order to maintain a sense of social control (Costa, 2018). In that sense, solutions for allocating HRI offered a sense of control over their audience, while retaining the benefits of sharing HRI as a means to gain motivation in their health work. However, using such strategies required technological skills and self-confidence—characteristics that not all participants had. Some of the younger participants did not approach HRI sharing as just dilemmic. They were united in their tendency to view boundaries between private and public information as ambiguous and to approach physical and digital social life as separate social spheres.

#### Embedding HRI

Concerns about losing control were also catalysts for other strategies that were used to resolve dilemmas. Several participants described how they would embed their HRI sharing on social media by using irony and humour. Articulating HRI using witty comments was a way of trying to avoid the risks of being perceived as lacking boundaries or as self-aggrandizing. This strategy consisted of creating a reflexive distance between the shared content and the sharing identity. A participant in his 40s laconically stated: “if I know myself, it will probably be more ironic” when reflecting upon how HRI content would be packaged. While irony seemed to be the most common way to create distance, the use of other expressions was also mentioned:

Well, I’m a bit of a jokey person, so I would’ve posted it [HRI] and I would have typed (…) a lot of different emojis and I would’ve written something like … “Here you can see what I need to work on”, or … and then a lot of training emojis and maybe someone like, you know that emoji that’s kind of blue in the face and has its hands on the cheeks, and just says “Aaaah!” like that. And then a little laughter (…) Things like that I often post a bit jokingly. (Woman in her 40s)

For participants, especially those who were avid social media users, using irony and humour seemed to be a way of trying to pre-empt potential conceptions before the perceived audience had the chance to label their content as *too* private, *too* sensitive, or *too* self-aggrandizing. By trying to convince potentially critical followers of their self-reflexive, humorous, and non-bragging, clearly bounded identities, they took control of any expected criticism that might threaten to disrupt their digital identity constructions. Hence, paradoxically, by distancing themselves from the digital identities that their HRI sharing would have implied had they not been ironic, they could also continue to enjoy the perks of those very identities—e.g., potential support or admiration, as well as likes and appreciative comments—without the risks that might otherwise have come with them (Glynos et al., 2015). This also means that the norm of individual responsibility within ideological discourses that proclaim health care via healthy lifestyles (Cheek, 2008), and its increasing connection with digital culture (Lupton, 2013), was given preference, successfully pre-empting and avoiding any processes of contestation.

## Discussion

The dilemmic approach to sharing HRI on social media (Cino & Formenti, 2021) furnished our analysis with tools to identify areas that were perceived as particularly troublesome and risky, and to explore how such concerns were managed and resolved. The participants’ identification of dilemmas and their strategies to resolve them were not actions they adhered to without reflection; the dilemmas were catalysts for conflicting feelings and practices, and participants’ negotiations indicative of what is at stake when people are being encouraged to share their HRI online.

Firstly, and confirming suggestions by Evans and Robertson (2020), *the arguments used in negotiations about what and how to share or not share were indebted to the social, cultural, and technological contexts in which participants were situated*. Participants who were invested in discourses that supported sharing and curated forms of self-representation did not generally see sharing of quite personal information as a crucial concern, but were still developing and using digital strategies to retain a sense of control. For them, developing such strategies came easily as they were familiar with how to activate settings to support their need for control. This enabled them to be open to using sharing as a vehicle to try to gain motivation in health work. Participants for whom sharing was not an integral part of their everyday digital social life, and who saw their social media profiles as direct representations of their offline identities, were more likely to find content such as HRI *too* sensitive and private to share on social media. This made sharing HRI into a tangible dilemma, and these participants were less inclined to utilize sharing on social media as a tool to become motivated for health work.

From this perspective, it can be argued that investment in discourses that support sharing and curated forms of self-representation unlock motivating affordances in social media sharing, while a lack of such investment prevents people from using social media as a method to gain motivation in health work. Lack of investment—and the feelings of suspicion and insecurity that came with it—seemed to enhance a perception of the delicacy of health-related social media content. In a sense, the perceived risks involved in sharing HRI turned platforms such as Facebook or Instagram into precarious social environments (Liliequist, 2020) that did not tease out feelings of motivation in any straightforward way. However, and importantly, lack of investment also entailed resistance towards socio-technological imperatives favouring sharing (Cino & Formenti, 2021; Lupton, 2016), leading participants to deliberately persist in keeping their HRI private and hold onto distinctions between private and public.

Hence, feelings of motivation do not emerge as innate qualities of individual subjects, as is sometimes implied in research about HRI sharing (e.g., Kye et al., 2019; Lin & Chang, 2018). Neither are they something that can be easily acquired with the right encouragement. Rather, both motivation and feelings of insecurity and loss of social control are co-produced with the social and technological circumstances within which they are enacted. They are also included as integral aspects of people’s self-identities and moral compasses. This result, we believe, reveals both opportunities and concerns for health and public health interventions that rely on peer-to-peer sharing of HRI. Hence, while people are generally open to sharing HRI in what are perceived as safe online environments (Kim & Choi, 2019), and while posting (and consuming) HRI seems to help encourage feelings of motivation in health work (Lin et al., 2018), it is a crucial insight that social and technological conditions can create ambiguities in sharing and discourage different groups and individuals from online practices through which such relations and feelings could be built.

Secondly, *participants’ suggested solutions to their perceived dilemmas had somewhat different social and technological underpinnings*. All the solutions can be seen as engaged in handling the distinctions between online and offline, which are often described as untenable in contemporary hybrid media culture (Hine, 2020; Lindgren, 2013). “Avoidance” was a solution that seemed to be informed by the entanglement of a morally charged desire to keep private and public separate, and unfamiliarity with using social media’s technological functions. This reinforced a sense of fear about what might happen once sensitive information is posted online. In a sense, the strategy of avoidance leads to missing out on the perks of the encouragement and motivation that sharing HRI can provide.

As solutions, “allocation” and “embedding” were very different from avoidance. They are both solutions that can be understood in terms of “selective self-presentation” (Liliequist, 2020) or “keeping privacy through effort” (Salte, 2023, p. 179; Marwick & boyd, 2014). Allocation was a strategy grounded in familiarity with social media technologies, and whole-hearted investment in imperatives that support sharing, as has been described by, among others, Van Dijck (2013) and Lupton (2016). Even people who used this strategy often argued in favour of maintaining boundaries between private and public but, because they were able to use their technological skills to adjust settings, they created a sense of control over their audiences. Allocation was thus a strategy that enabled engagement with social media communities within which support for lifestyle change can be provided.

The solution that used ironic *embeddings* to control followers’ perceptions of health-related posts by embedding them in ironic or humorous comments may be seen as emanating from and serving different social ideologies. The strategy was sometimes just a way to create distance between oneself and one’s post, so as to save oneself from unwanted interpretations. But this strategy was sometimes also informed by a type of cultural criticism that saw public interventions as forceful governmentality that imposed feelings of imperfection and shame on citizens. Irony in particular brings ambiguity to the shared content, in that it both problematizes and shifts the meaning of the message. It has been suggested that such ambiguities constitute resistance against dominant ideological discourses of healthiness as an uncontested goal (e.g., Glynos, 2008). In that sense, the strategy of embedding destabilizes norms about healthiness as an important individual goal and responsibility. Through its articulation via irony or humour, shared HRI may instead become an expression of *non-engagement* on the part of the poster. While this interpretation may certainly contribute to resistance towards healthiness norms, it may just as well contribute to the naturalization of HRI as normalized social media content, since it does, in fact, involve sharing HRI online. In both cases, the solution of embedding HRI in irony and humour may indeed confound the goals of health and public health interventions, which are increasingly looking towards social media as a way forward.

### Reclaiming agency

It is important to remember that the solutions described above were not neutral efforts to resolve external dilemmas. As suggested by Bauman (2007) and others (e.g., Friesen, 2017; Fulton & Kibby, 2017), online strategies are also “technologies of the self” (Foucault, 1988). Seen in this way, participants’ strategies for handling perceived dilemmas are not only the work of stable subjects who are trying to control their self-presentations online. These strategies also *transform* the identities of the authors of the posts (Buongiorno, 2019). This particular aspect has proved key to understanding why sharing strategies within interventions work better for some than for others. It is particularly important to reflect upon how, for example, both non-engagement and ironic engagement may indeed produce subjects who are positioned in opposition to the goals that health and public health interventions are aiming to achieve, hence attesting to the existence of ambiguities about the ways in which the sharing of HRI supports health work (Giustini et al., 2018).

At an overarching level, the suggested solutions were produced under circumstances in which participants were involved in an intervention practice that—like most interventions—aimed to educate people and govern lifestyles. This is a classic form of population-based biopolitics upon which many interventions are based (Tengland, 2012), but which, when used via social media, have been claimed to erode patient trust (Donnelly, 2023). In cases where actors in health and public health are also looking to encourage people to use their own personal social media to accomplish results, the intervention practice encourages participants to transgress boundaries between private and public at many levels. Being representatives of the public healthcare system, the intervention asks permission to enter the private worlds of participants. At the same time, it asks participants to share private health data online to an audience that is perceived by some as public and unknown (Van Dijck & Poell, 2016). Their reason for doing so is of course the results of studies that attest to efficiency regarding health results (e.g., Maher et al., 2014; Sawesi et al., 2016). But the transgression between public and private that is being encouraged is also the main reason why the participants identified dilemmas—and their subsequent efforts to resolve them through particular and, in terms of health promotion, possibly counterproductive practices.

People’s willingness to share health- and lifestyle-related personal information is a critical factor when utilizing social media in health and public health work. As interventions are increasingly trying to make use of sharing on social media to achieve results (Abroms, 2019; Chen et al., 2019; Zhang et al., 2020), their thinking is no doubt underpinned by notions that digital media have become incorporated into people’s daily lives. While this was certainly true for most of our study participants, their familiarity with social media, their skills in adjusting settings, and their subsequent feelings of security online, all differed. Taking into account the above interpretation of interventions as biopolitical and transgressive practices, the solutions suggested by the participants can be understood as efforts to reclaim agency in a situation where control is perceived as being elusive.

### Preaching to the choir?

A social and cultural perspective on the uses made by health interventions of people’s personal sharing practices showed that sharing HRI on social media involved significant dilemmas, but that participants’ responses to these dilemmas were never a question of *either* accepting *or* resisting (Salte, 2023). Rather than simply technological ignorance or unfamiliarity with social media, the dilemmas included social notions of privacy and identity that comprised the uncertainties involved in participants’ relations to social media. Because of this, it is likely that the perceived dilemmas are not easily resolved by “nudging” or educational efforts, strategies that have been suggested and used to increase people’s engagement in online interventions (e.g., Azzopardi-Muscat & Sørensen, 2019; Purohit et al., 2023). Not only the dilemmas themselves, but also the strategies that were used to resolve them, appeared to be potentially counterproductive, creating meaning around health work that either did not reach the parts of people’s networks that would benefit from it, or constituted health work as non-serious and unimportant.

Participants identified negative triggering effects on others as an important reason for not sharing their HRI. There were also lingering associations between disclosing HRI and exhibitionism and the inability to draw a line between private and public, which transferred itself to the poster’s identity. The fear of becoming “one of those” who shared *too* much, and information that was *too* private was frequently mentioned. That such a transformation posed a real threat was supported by the equally frequent references to others in the participants’ networks who were defined by this type of derogatory description.

Given the above, it is important that the different strategies used for sharing and not sharing information—avoidance, allocation, embedding—are approached, not as matters to be overcome, but rather as important indicators of what is at stake in public health interventions—especially when such interventions find their way into people’s personal networks. Bearing this in mind, seeing social media as a quick fix for developing engagement in health and healthy lifestyles might be “preaching to the choir”—only reaching groups who are devoted social media users and inclined to use sharing to gain and maintain motivation. For others, such as older people, the notion of online sharing might generate important motivation for health work, but it might also give rise to feelings of insecurity, as well as to criticism and distancing from the benefits of such work. Although older people are a heterogeneous group and are increasingly approaching social media and peer-to-peer sharing as an inherent part of everyday social life (Chew et al., 2023; Cotten et al., 2022), it has been argued that they tend to be less interested in engaging with social media and social media sharing than younger people (Newman et al., 2021). The main arguments used by older age groups for not using social media are lack of familiarity and skill in relation to different social media systems, and also privacy issues and the perceived triviality and self-centeredness of social media interactions (Jung et al., 2017; Lüders & Brandtzæg, 2017; Wilson et al., 2023).

Attempting not to persuade people, but to address conflicting feelings through the design of interventions and the technologies used, can be key to health initiatives involving the sharing of HRI. So can an understanding of the complex connections with identity, morals, and social relations that characterize social media practices. But if peer-to-peer sharing is to be used in interventions at the community and population levels, the different strategies people use to avoid and/or control HRI disclosures also illuminate the need to acknowledge groups that do not want to share on social media or see any benefits from doing so. Our results suggest that there is reason to reflect upon the effects of interventions that rely heavily on people’s social media sharing; while, for some, sharing HRI with allocated groups seems to increase their motivation to engage in health-related work, others still contest online sharing practices. Hence, to ensure equality in health promotion, health and public health initiatives need to be careful to avoid the sometimes counterproductive effects this may have on some people’s engagement.
